# Impact of high Spirulina diet, extruded or supplemented with enzymes, on blood cells, systemic metabolites, and hepatic lipid and mineral profiles of broiler chickens

**DOI:** 10.3389/fvets.2024.1342310

**Published:** 2024-03-26

**Authors:** Maria P. Spínola, Cristina M. Alfaia, Mónica M. Costa, Rui M. A. Pinto, Paula A. Lopes, José M. Pestana, João C. Tavares, Ana R. Mendes, Miguel P. Mourato, Beatriz Tavares, Daniela F. P. Carvalho, Cátia F. Martins, Joana I. Ferreira, Madalena M. Lordelo, José A. M. Prates

**Affiliations:** ^1^CIISA - Centro de Investigação Interdisciplinar em Sanidade Animal, Faculdade de Medicina Veterinária, Universidade de Lisboa, Lisbon, Portugal; ^2^Laboratório Associado para Ciência Animal e Veterinária (AL4AnimalS), Lisbon, Portugal; ^3^JCS, Laboratório de Análises Clínicas Dr. Joaquim Chaves, Avenida General Norton de Matos, Algés, Portugal; ^4^iMED.UL, Faculdade de Farmácia, Universidade de Lisboa, Avenida Professor Gama Pinto, Lisbon, Portugal; ^5^Instituto Superior de Agronomia, Universidade de Lisboa, Lisbon, Portugal; ^6^LEAF - Linking Landscape, Environment, Agriculture and Food, Instituto Superior de Agronomia, Universidade de Lisboa, Associated Laboratory TERRA, Lisbon, Portugal

**Keywords:** feed enzymes, hepatic compounds, microalga extrusion, plasma metabolites, poultry nutrition

## Abstract

The impact of 15% dietary inclusion of Spirulina (*Arthrospira platensis*) in broiler chickens was explored, focusing on blood cellular components, systemic metabolites and hepatic lipid and mineral composition. From days 14 to 35 of age, 120 broiler chickens were divided and allocated into four dietary treatments: a standard corn and soybean meal-based diet (control), a 15% Spirulina diet, a 15% extruded Spirulina diet, and a 15% Spirulina diet super-dosed with an enzyme blend (0.20% porcine pancreatin plus 0.01% lysozyme). The haematological analysis revealed no significant deviations (*p* > 0.05) in blood cell counts across treatments, suggesting that high Spirulina inclusion maintains haematological balance. The systemic metabolic assessment indicated an enhanced antioxidant capacity in birds on Spirulina diets (*p* < 0.001), pointing toward a potential reduction in oxidative stress. However, the study noted a detrimental impact on growth performance metrics, such as final body weight and feed conversion ratio (both *p* < 0.001), in the Spirulina-fed treatments, with the super-dosed enzyme blend supplementation failing to alleviate these effects but with extrusion mitigating them. Regarding hepatic composition, birds on extruded Spirulina and enzyme-supplemented diets showed a notable increase in *n*-3 fatty acids (EPA, DPA, DHA) (*p* < 0.001), leading to an improved *n*-6/*n*-3 PUFA ratio (*p* < 0.001). Despite this positive shift, a reduction in total hepatic lipids (*p* = 0.003) was observed without a significant change in cholesterol levels. Our findings underscore the need for further exploration into the optimal inclusion levels, processing methods and potential enzymatic enhancements of Spirulina in broiler diets. Ultimately, this research aims to strike a balance between promoting health benefits and maintaining optimal growth performance in poultry nutrition.

## 1 Introduction

The increasing global quest for sustainable and eco-friendly protein sources, especially in poultry farming, has driven the exploration toward unconventional feed components. Among these, microalgae, particularly those from the Spirulina genus, have emerged as noteworthy contenders (1–3). *Arthrospira platensis*, ubiquitously known as Spirulina, is a cyanobacterium heralded for its superior protein content, carbohydrates, essential fatty acids, vitamins, pigments and diverse mineral profile. However, these contents are subject to modulation based on cultivation conditions and seasonal variations (4, 5).

Apart from its robust nutritional portfolio, Spirulina exhibits a range of functional benefits including antimicrobial, antioxidant, anti-inflammatory and immunomodulatory properties (5–7). However, the alga’s predominantly indigestible cell wall, resembles the Gram-negative bacteria with a composition mostly of peptidoglycan and lipopolysaccharides, presenting digestibility challenges to monogastric animals (8). Moreover, the bioaccessibility of algal proteins can be thwarted due to the protein-pigment complexes, such as phycocyanins interfaced with thylakoid membranes (9).

Although carbohydrases, like lysozyme, have shown promise in partially dismantling the Spirulina cell wall (10), a gelation process appears to ensnare nutrients, impeding their digestion (3, 11). Preliminary attempts to hydrolyse Spirulina proteins through physical pre-treatments coupled with pancreatin or trypsin have been made (12, 13), but the realm of deploying such enzymes to degrade proteins in *A. platensis* feed for monogastric animals remains largely unexplored. However, emerging studies accentuate the potential efficacy of specific exogenous enzymes, such as carbohydrate-active enzymes, in breaching the Spirulina cell wall and amplifying the bioavailability of its nutritional benefits (14). The incorporation of these exogenous enzymes as feed additives in poultry diets is a well-trodden strategy to mitigate the paucity of endogenous enzymes and neutralize anti-nutritional factors, enhancing the digestibility of dietary constituents (15, 16). Furthermore, these enzymes foster the generation of prebiotic oligosaccharides, thus buoying gut health and growth parameters (17).

Traditionally, microalgae have been utilized more as supplements than core ingredients in monogastric diets (18). Preceding inquiries from this team evaluated the ramifications of integrating microalgae as a feed ingredient on growth performance, health status, and hepatic lipid composition in broilers (19, 20), and extended to pigs (19, 20). This investigation pioneers the examination of the synergistic effects of prolonged feeding with a high level of *A. platensis*, either alone or supplemented with different enzymes (predominantly peptidases, EC 3.4), on the health and hepatic lipid metabolism in broilers. While the positive impacts of dietary Spirulina supplementation on broiler performance and health have been tentatively probed [e.g., Pestana et al. (3)], a realm unexplored persists concerning the interactive effects of super-dosing multienzymes in conjunction with higher Spirulina inclusion levels. Indeed, previous reports showed the potential of super-dosed (higher than recommended) multi-enzymes to improve growth performance, gut health, and nutrient absorption in poultry (21–23). The application of extruded Spirulina in one of the dietary treatments seems to be a promising combination, as shown in *in vitro* studies, to enzymatic pre-treatments (e.g., porcine pancreatin), as the high pressure and temperature may cause a disruption in microalga’s cell wall, contributing to improving the bioavailability of relevant proteins, such as phycocyanin (12, 13).

The purpose of this work was to assess the impact of incorporating 15% *A. platensis* biomass in broiler diets, either exclusively, or in combination with a super-dosing enzyme blend (pancreatin and lysozyme), or when Spirulina is extruded, from day 14 to 35, on broilers’ blood cells, plasma metabolites, and hepatic composition, comprising fatty acids, antioxidant diterpenes, pigments and mineral profile.

## 2 Materials and methods

### 2.1 Animal housing and dietary treatments

The research enlisted a group of one hundred and twenty, one-day-old male Ross 308 broiler chicks acquired from Pinto Valouro (Bombarral, Portugal), each exhibiting an average initial weight of 39.3 ± 2.30 g. From day 1, they were randomly lodged in 40 wire-netted enclosures (56 cm height x 56 cm length x 50 cm width) for 35 days under regulated environmental conditions. Ambient temperature and airflow were persistently supervised as per the previously established protocols (2, 19). Adhering to the 3R’s doctrine (Reduce, Refine and Replace) to curtail the number of animals employed, every enclosure hosted three chicks, with 10 identical enclosures designated for each dietary experiment. The experimental trial was conducted in the School of Agriculture at the University of Lisbon (Lat: 38°42′27.5”N; Lng: 9°10′56.3”W). The Animal Welfare Committee of the School of Agriculture at the University of Lisbon (ORBEA/ISA) approved all the experimental methodologies involving animals, allocating the study a protocol code number 0421/000/000/2022.

In the initial fortnight, chicks were administered a staple diet formulated from corn and soybean meal sourced from Rações Veríssimo S. A. (Leiria, Portugal). Following this phase, from day 14 through day 35, they transitioned to one of four distinct experimental diets furnished *ad libitum* daily. These diets encompassed: (1) a standard diet predicated on corn and soybean meal (CTR); (2) a diet fortified with 15% Spirulina powder (procured from Allmicroalgae, Pataias, Portugal) (SP); (3) a diet containing 15% extruded Spirulina powder (SPE); and (4) a diet supplemented with 15% Spirulina powder alongside a tailored enzyme blend, constituted by 0.20% porcine pancreatin extract (sourced from Merck, Darmstadt, Germany) and 0.01% lysozyme extracted from chicken egg white (sourced from Sigma-Aldrich, Missouri, United States) (SPM). The microalga extrusion was performed by Sparos company (Olhão, Portugal), following detailed conditions: 340 mL of water addition per minute, at 34 bars and 118°C for the last extrusion barrel. This procedure occurred from 3 to 7 s. Then, the algal pellets were dried for 8 and 10 min at 120°C (12, 13). The encapsulated porcine pancreatin extract comprised 350 FIP-U/g of protease, 6,000 FIP-U/g of lipase, and 7,500 FIP-U/g of amylase. Lysozyme powder contained 70,000 U/mg protein. A detailed composition of the feed constituents and additives for each experimental diet is provided in Table 1. During the trial, a 2.5% mortality was observed (three animals of 120 in total).

**Table 1:** Ingredient composition of broiler experimental diets from day 14 to day 35 (%, as fed basis).

	CTR	SP	SPE	SPM
Corn	55.4	62.2	63.7	62.2
Soybean meal	36.9	18.6	17.3	18.6
Sunflower oil	4.10	1.00	0.80	1.00
Sodium chloride	0.40	0.40	0.41	0.40
Calcium carbonate	1.08	1.39	1.38	1.39
Dicalcium phosphate	1.60	0.90	0.92	0.90
DL-Methionine	0.12	0.03	0.04	0.03
L-Lysine	0.00	0.05	0.10	0.05
Vitamin-mineral premix^1^	0.40	0.40	0.40	0.40
*Arthrospira platensis* powder	0.00	15.0	0.00	15.0
*Arthrospira platensis* extruded powder	0.00	0.00	15.0	0.00
Enzyme mixture	0.00	0.00	0.00	0.21

CTR, corn and soybean meal-based diet; SP, 15% Spirulina; SPE, 15% extruded Spirulina; SPM, 15% Spirulina + 0.21% Enzyme mixture (0.20% porcine pancreatin + 0.01% lysozyme).

^1^Premix provided the following per kilogram of diet: pantothenic acid 10 mg, vitamin D_3_ 2,400 IU, cyanocobalamin 0.02 mg, folic acid 1 mg, vitamin K_3_ 2 mg, nicotinic acid 25 mg; vitamin B_6_ 2 mg, vitamin A 10000 UI, vitamin B_1_ 2 mg, vitamin E 30 mg, vitamin B_2_ 4 mg, Cu 8 mg, Fe 50 mg, I 0.7 mg, Mn 60 mg, Se 0.18 mg, Zn 40 mg.

Weekly assessments were conducted to record the weights of broilers and feeders, with daily feed provisioning to compute the parameters of body weight gain (BWG) (ascertained by the weekly weight differential divided by 7), average daily feed intake (ADFI) (calculated as weekly consumption per cage, divided by 7) and the feed conversion ratio (FCR) (the quotient of weekly consumption divided by 3 and weekly body weight gain).

### 2.2 Analysis of microalga and experimental diets

Table 2 shows the proximal composition of the microalga and the experimental diets offered to broilers from day 14 to 35. The assessment of dry matter, crude protein, crude fat, and ash content was conducted in adherence to the methodologies stipulated by AOAC (24). The quantification of gross energy content within both the microalga and the diets was executed employing an adiabatic bomb calorimetry (Parr 1,261, Parr Instrument Company, Moline, IL).

**Table 2:** Proximal and nutrient compositions of *Arthrospira platensis* and experimental diets offered to broilers from day 14 to 35.

	Spirulina	Extruded Spirulina	CTR	SP	SPE	SPM
Gross energy (kcal/kg as dry matter)	4,476	4,504	4,600	4,418	4,390	4,413
Proximate composition (% as dry matter)						
Dry matter	94.8	98.4	87.9	88.4	89.0	88.7
Crude protein	52.6	53.7	24.1	23.0	23.5	23.5
Crude fat	6.56	5.81	7.56	5.15	4.82	5.06
Ash	21.6	21.3	6.29	8.19	8.44	8.47
Fatty acid composition (% total fatty acids)						
14:0	0.43	0.39	0.072	0.11	0.11	0.11
16:0	39.7	49.5	9.74	20.2	16.7	17.2
16:1*c*9	4.95	4.34	0.12	0.73	0.87	0.81
17:0	0.41	0.45	0.063	0.15	0.13	0.12
17:1*c*9	1.88	1.60	0.00	0.22	0.29	0.24
18:0	1.75	1.68	3.41	3.12	2.32	2.62
18:1*c*9	6.34	5.32	29.1	25.0	26.6	25.9
18:2*n*-6	17.8	15.0	52.9	43.0	45.6	45.4
18:3*n*-3	15.8	12.0	1.15	2.94	3.46	3.30
20:0	0.189	0.20	0.53	0.67	0.43	0.52
20:1*c*11	0.91	0.029	0.19	0.20	0.21	0.20
22:0	0.247	0.29	0.99	0.65	0.35	0.53
Diterpene profile (μg/g)						
α-Tocopherol	27.2	29.5	47.5	29.1	24.4	34.7
α-Tocotrienol	n.d.	n.d.	4.59	3.52	4.12	3.80
β -Tocopherol	n.d.	n.d.	0.55	0.35	0.30	0.35
γ-Tocopherol + β-tocotrienol	n.d.	n.d.	6.44	5.59	6.47	5.86
γ-Tocotrienol	n.d.	n.d.	n.d.	n.d.	n.d.	n.d.
Pigments (μg/g dry matter)						
Chlorophyll-*a* (Ca)^1^	866	1,467	13.3	472	209	494
Chlorophyll-*b* (Cb)^2^	191	1,189	23.5	91.7	167	81.2
Σ Chlorophylls (Ca + b)^3^	1,057	2,656	36.8	564	376	575
β-Carotene	243	78.0	0.96	48.4	23.9	55.0
Σ Carotenoids (Cx + c)^4^	243	154	3.06	117	43	126
Σ Chlorophylls and carotenoids (Ccc)^5^	1,300	2,810	39.8	681	419	701
Minerals (mg/kg dry matter)						
Calcium (Ca)	42.8	32.2	13.5	16.7	16.9	17.5
Magnesium (Mg)	3.06	3.21	2.21	1.91	2.00	2.04
Phosphorous (P)	11.4	11.6	8.18	6.87	7.37	7.49
Potassium (K)	22.9	19.7	11.0	9.77	10.1	10.3
Sodium (Na)	34.2	25.7	5.55	8.85	9.60	9.41
Sulphur (S)	8.43	8.03	3.30	3.27	3.50	3.48
Copper (Cu)	0.010	0.006	0.014	0.013	0.012	0.010
Iron (Fe)	0.90	0.89	0.15	0.23	0.25	0.25
Manganese (Mn)	0.094	0.083	0.090	0.071	0.073	0.086
Zinc (Zn)	0.064	0.064	0.087	0.075	0.086	0.089

CTR, corn and soybean meal-based diet; SP, 15% Spirulina; SPE, 15% extruded Spirulina; SPM, 15% Spirulina +0.21% Enzyme mixture (0.20% porcine pancreatin +0.01% lysozyme).

n.d., not detected.

^1^Ca = 11.24 × A662 nm - 2.04 × A645 nm.

^2^Cb = 20.13 × A645 nm - 4.19 × A662 nm.

^3^Ca + b = 7.05 × A662 nm + 18.09 × A645 nm.

^4^Cx + c = (1,000 × A470 nm - 1.90 × Ca - 63.14 × Cb) / 214.

^5^Ccc = (Ca + b) + (Cx + c).

The fatty acid composition in *A. platensis* and the experimental diets was done after a singular extraction and acidic methylation step, aligning with the method delineated by Sukhija and Palmquist (25). The resultant fatty acid methyl esters (FAME) were analyzed utilizing gas chromatography interfaced with a flame ionization detector (HP7890A Hewlett-Packard, Avondale, PA), deploying a Supelcowax® 10 capillary column (30 m × 0.20 mm i.d., 0.20 μm film thickness, Supelco, Bellefonte, PA, United States), by the protocol advanced by Alfaia et al. (26). Identification of FAME within the samples was achieved through juxtaposition with a standard FAME mixture (37 Component FAME mix C4-C24, Supelco, Bellefonte, PA, USA). The heneicosanoic acid (21.0) methyl ester was enlisted as the internal standard for the quantification of individual fatty acids, with the fatty acid content articulated as a percentage of the total fatty acids.

The diterpenes and β-carotene within the experimental diets were assessed by adhering to the methodology delineated by Prates et al. (27). Duplicated samples, each weighing 0.1 g, were fortified with ascorbic acid (vitamin C) and underwent a saponification reaction in a water bath maintained at 80°C for 15 min. Following extraction and centrifugation (2,500 rpm, 10 min), the *n*-hexane phases were scrutinized utilizing an HPLC system (Agilent 1,100 Series, Agilent Technologies Inc., Palo Alto, CA, United States) equipped with a normal-phase silica column (Zorbax RX-Sil, 5 μm particle size, 250 mm × 4.6 mm i.d.). β-carotene was discerned employing a UV–visible photodiode array detector (*λ* = 450 nm), while tocopherols and tocotrienols were identified with a fluorescence detector (excitation *λ* = 295 nm and emission *λ* = 325 nm). The quantification of β-carotene and analogs of vitamin E was facilitated through the utilization of standard curves correlating peak area to concentration.

The pigment profile of the experimental diets was ascertained with minor alterations to the protocols expounded by Hynstova et al. (28) and Pestana et al. (3). Concisely, 0.1 g of samples were amalgamated with 5 mL of acetone, and homogenized in the dark, followed by centrifugation (2,500 rpm, 10 min) the supernatant was isolated and analysed. Chlorophylls *a* and *b* were detected at wavelengths of 662 nm and 645 nm, respectively, and total carotenoids at 470 nm using UV–Vis spectrophotometry (Genesys 150, Thermo Scientific, Waltham, Massachusetts, United States). The pigment content was deduced employing equations provided by Hynstova et al. (28).

The mineral composition of *A. platensis* and experimental diets was deciphered by allocating 0.3 g of samples into a digestion tube, introducing nitric acid (65%) and hydrochloric acid (37%), and digesting at 95°C within a ventilated chamber for 16 h. Subsequently, hydrogen peroxide (30%) was added as outlined by Ribeiro et al. (29). The resultant solution was filtered and analysed employing Inductively Coupled Plasma - Optical Emission Spectrometry (ICP-OES, iCAP 7,200 duo Thermo Scientific, Waltham, MA, United States), with mineral quantification achieved through standards and calibration curves.

### 2.3 Slaughtering and sample collection

At the end of the trial, in a room with specific conditions for slaughtering, one 35-day-old broiler, medium weight, from each pen was selected, for slaughter, using electrical stunning followed by manual exsanguination. Blood samples were collected (10 mL), on the same day, directly into Sarstedt tubes (Numbrecht, Germany), and were subsequently centrifuged to obtain plasma. Liver samples were collected, minced, vacuum-sealed, and stored at −20°C for future analysis. The remaining birds were euthanized as described above.

### 2.4 Analysis of blood cells and plasma metabolites

As articulated by Madeira et al. (19), red blood cells, white blood cells, and thrombocytes were enumerated employing Sysmex XN-10 analysers (Sysmex Corporation, Kobe, Japan). The red blood cell tally was determined via the impedance variation method, after hydrodynamic focusing. Differential enumeration of white blood cells (%) was executed on blood smears, which were decolourized utilizing the May-Grünwald-Giemsa technique. Haemoglobin concentration was gauged photometrically, with sodium lauryl sulphate serving as the reagent.

Selected plasma metabolites, encompassing glucose, urea, creatinine, total protein and assorted lipid forms (triacylglycerols, TAG; total cholesterol; HDL-cholesterol; and LDL-cholesterol), in addition to enzymatic activities of alanine aminotransferase (ALT, EC 2.6.1.2), aspartate aminotransferase (AST, EC 2.6.1.1), alkaline phosphatase (ALP, EC 3.1.3.1) and γ-glutamyltransferase (GGT, EC 2.3.2.13) were ascertained employing diagnostic kits from Roche Diagnostics’ Modular Hitachi Analytical System (Mannheim, Germany). Formulas delineated by Friedewald et al. (30) and Covaci et al. (31) were applied for the computation of VLDL-cholesterol and total lipids, respectively. C-reactive protein levels were evaluated via immunoturbidimetry (Roche Diagnostics, Meylan, France). Primary electrolytes including Na^+^, K^+^ and Cl^−^ were appraised through indirect potentiometry.

### 2.5 Analysis of total lipids and fatty acid composition in the liver

Total lipids were isolated from the lyophilized liver specimens, in duplicate, employing a solvent mixture of dichloromethane and methanol (2:1 v/v), and quantified via gravimetric analysis as per the protocol delineated by Folch et al. (32). The fatty acid composition within the liver specimens was deciphered akin to the methodology applied for *A. platensis* and the experimental diets. Nonetheless, a sequential process of alkaline followed by acidic transesterification was leveraged to convert the fatty acids into FAME, diverging from the sole acidic methylation procedure. The chromatographic conditions were congruent with the protocol formerly articulated by Alfaia et al. (26).

### 2.6 Determination of total cholesterol, diterpenes, and β-carotene in the liver

Duplicate liver specimens, each weighing 0.75 g, were employed for the extraction of total cholesterol, diterpenes and β-carotene, adhering to the identical procedure delineated for the experimental diets. This encompassed direct saponification, singular n-hexane extraction, and subsequent HPLC analysis as per the protocol detailed in Prates et al. (27).

For pigment extraction (encompassing chlorophyll-*a*, chlorophyll-*b*, and total carotenoids) from liver specimens, a slightly adapted version of the method deployed for the experimental diets was utilized. In brief, 1.5 g of liver tissue was amalgamated with 3 mL of acetone, followed by a 1-min homogenization in a darkened environment. Post-centrifugation (3,000 rpm for 5 min at 4°C), the supernatant was sequestered and examined under the conditions stipulated by Coelho et al. (33). The pigment quantities were ascertained utilizing the equations presented by Hynstova et al. (28).

### 2.7 Determination of mineral profile in the liver

The hepatic mineral profile was gauged following the established procedure for the microalga and diets, except for bromide, whose determination was conducted as per the method expounded by Delgado et al. (34).

### 2.8 Statistical analysis

The dataset was subjected to analysis as a completely randomized design employing the Generalized Linear Mixed (GLM) model within the framework of the Statistical Analysis System (SAS) software (SAS Institute Inc., Cary, NC) (35). The experimental diet was the single fixed factor and the primary effect within the statistical model. The experimental unit was either the enclosure or the animal depending on the parameters under evaluation. The enclosure was designated for assessing body weight, body weight gain, average daily feed intake, and feed conversion ratio, while the individual bird was the unit of analysis for the remaining parameters (plasma metabolites, and comprehensive hepatic parameters). The data was tested for the homogeneity of variances using Levene’s test, and for normality using the Shapiro–Wilk test. For multiple comparisons among least squares means, the adjusted Tukey–Kramer approach (PDIFF option) was invoked. All values were articulated as the mean along with the SEM (standard error of the mean). A threshold of *p*-values less than 0.05 was established for the determination of statistical significance.

To explore the interrelations between blood parameters and hepatic variables, a Principal Component Analysis (PCA) was conducted utilizing the SPSS Statistics for Windows software suite (IBM Corp., 2020 release, version 27.0, Armonk, NY, United States).

## 3 Results

### 3.1 Effect of experimental diets on birds’ performance and feed intake

**Figure 1:**
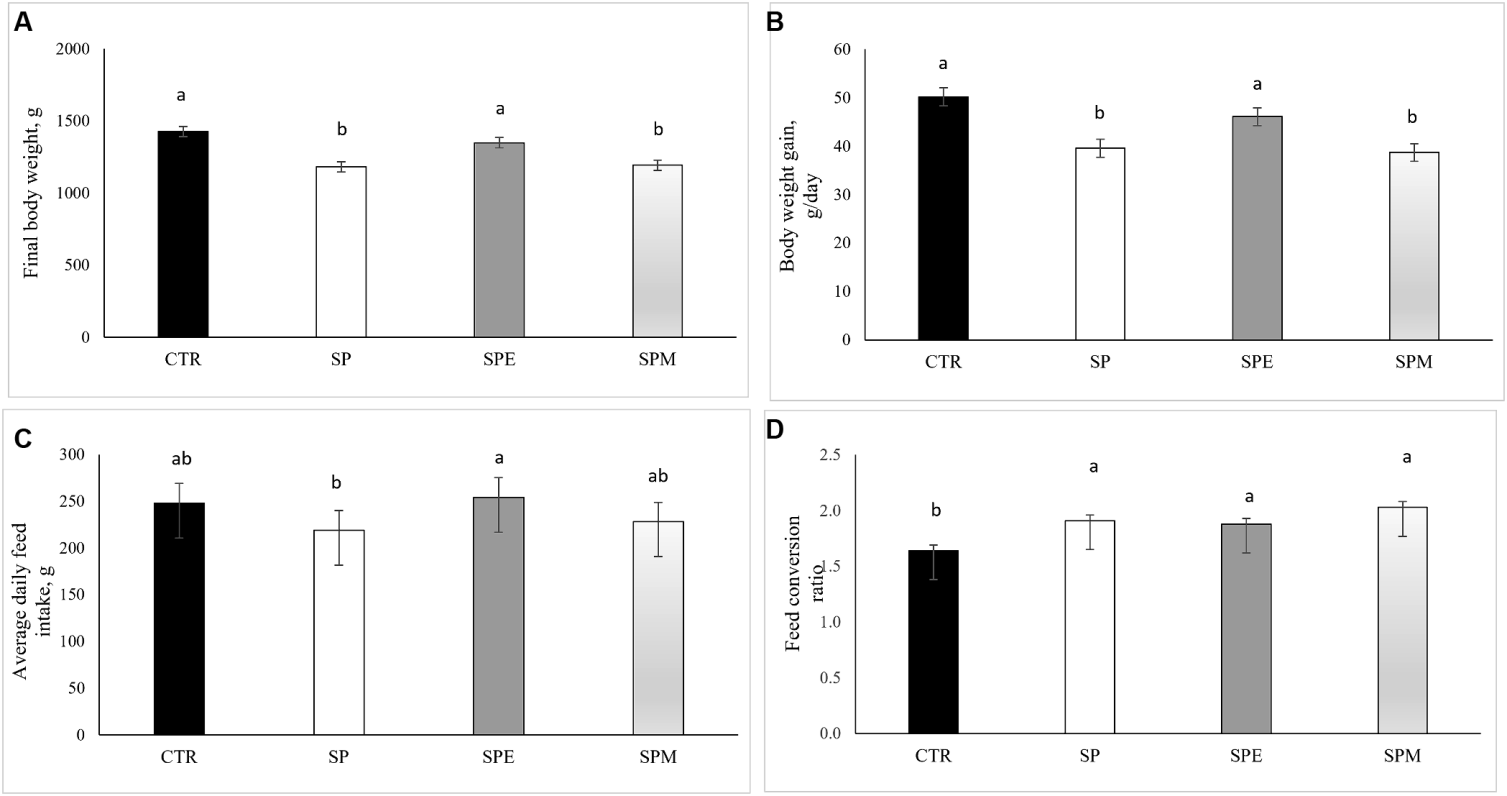
Impact of dietary treatments on broilers´ growth performance: **(A)** Final body weight (g); **(B)** Body weight gain (g/day); **(C)** Average daily feed intake (g); **(D)** Feed conversion ratio. CTR, corn and soybean meal-based diet; SP, 15% Spirulina; SPE, 15% extruded Spirulina; SPM, 15% Spirulina +0.21% enzyme mixture (0.20% porcine pancreatin +0.01% lysozyme). Values are shown as mean ± SEM (standard error of the mean). ^a,b,c^Different superscripts within a row indicate a significant difference (*p* < 0.05).

The investigation into the growth performance of broiler chickens under different treatments is presented in Figure 1 for contextual purposes. This assessment covered the period from days 14 to 35 of the chickens’ development. The findings showed that diets containing solely Spirulina (SP) and Spirulina combined with the enzyme mix (SPM) significantly reduced the final body weight (BW) of the birds, compared to the control diet. However, no significant differences in final BW were observed between the group fed extruded Spirulina (SPE) and the control group. A similar pattern emerged in the body weight gain (BWG) metrics, with the SPE diet showing results comparable to the control group, distinctly different from the SP and SPM diets. In terms of average daily feed intake (ADFI), an interesting increase was noted in the SPE group compared to the SP diet. Regarding the feed conversion ratio (FCR), all groups incorporating Spirulina led to an increase in FCR relative to the control, signifying a decrease in feed efficiency.

### 3.2 Effect of experimental diets on haematological and biochemical profile

Blood cell count and plasma metabolites from broilers fed 15% of *A. platensis*, either exclusively, or when Spirulina is extruded or combined with an enzyme blend (pancreatin and lysozyme) are displayed in Table 3. The counts for white blood cells (*p* = 0.702), granulocytes (*p* = 0.769), lymphocytes (*p* = 0.741), monocytes (*p* = 0.473), red blood cells (*p* = 0.377), thrombocytes (*p* = 0.708), and also haemoglobin (*p* = 0.062) were not affected by diets.

**Table 3:** Haematological and biochemical profiles of broilers fed the experimental diets.

	CTR	SP	SPE	SPM	SEM	*P*-value
White blood cells (×10^9^/L)	5.50	8.22	4.98	6.92	2.15	0.702
Granulocytes (%)	43.3	45.8	42.4	47.3	4.77	0.769
Lymphocytes (%)	53.7	53.0	56.6	51.7	4.28	0.741
Monocytes (%)	1.33	1.00	1.00	1.00	0.192	0.473
Red blood cells (×10^12^/L)	2.93	3.55	3.79	3.39	0.368	0.377
Haemoglobin (g/dL)	9.22	10.1	10.2	9.82	0.276	0.062
Thrombocytes (×10^9^/L)	22.7	22.2	25.6	23.7	2.23	0.708
Glucose (mg/dL)	248^b^	251^ab^	256^ab^	260^a^	2.78	0.021
Urea (mg/dL)	1.88^a^	1.60^b^	1.27^c^	1.90^a^	0.071	<0.001
Creatinine (mg/dL)	0.008^b^	0.009^b^	0.018^a^	0.016^a^	0.002	<0.001
Cholesterol (mg/dL)	96.5^b^	114^a^	112^a^	98.4^b^	2.07	<0.001
LDL-cholesterol (mg/dL)	12.4^b^	22.5^a^	20.3^a^	15.1^b^	0.730	<0.001
HDL-cholesterol (mg/dL)	71.4^a^	67.6^ab^	70.9^ab^	66.6^b^	1.24	0.020
VLDL-cholesterol (mg/dL)	9.70^d^	18.3^a^	15.6^b^	12.4^c^	0.648	<0.001
Triacylglycerols (mg/dL)	48.5^d^	91.4^a^	78.1^b^	62.0^c^	3.24	<0.001
Total lipids (mg/dL)	392^b^	468^a^	451^a^	409^b^	5.28	<0.001
ALT (U/L)	1.10	1.20	1.20	1.30	0.131	0.763
AST (U/L)	245^a^	180^b^	175^b^	163^b^	6.08	<0.001
GGT (U/L)	22.1^bc^	26.6^a^	24.2^ab^	19.3^c^	1.04	0.002
ALP (U/L)	1581^b^	1705^a^	1543^b^	1493^b^	24.9	<0.001
Total protein (g/dL)	2.44	2.80	2.50	2.54	0.152	0.358
C-reactive protein (mg/dL)	0.008^a^	0.008^a^	0.002^b^	0.007^a^	0.0013	0.002
Na^+^ (mEq/L)	148^a^	148^a^	151^a^	144^b^	0.818	<0.001
K^+^ (mEq/L)	6.51	6.28	5.84	6.45	0.204	0.104
Cl^−^ (mEq/L)	111^ab^	110^b^	113^a^	105^c^	0.803	<0.001

CTR: corn and soybean meal-based diet; SP: 15% Spirulina; SPE: 15% extruded Spirulina; SPM: 15% Spirulina +0.21% Enzyme mixture (0.20% porcine pancreatin +0.01% lysozyme).

TAG, triacylglycerols; HDL, high-density lipoproteins; LDL, low-density lipoproteins; VLDL, very low-density lipoproteins; ALT, alanine aminotransferase (EC 2.6.1.2); AST, aspartate aminotransferase (EC. 2.6.1.1); ALP, alkaline phosphatase (EC 3.1.3.1); GGT, glutamyltransferase (EC 2.3.2.13).

Total lipids = [total cholesterol] × 1.12 + [TAG] × 1.33 + 148, as described by Covaci et al. (31).

VLDL-CHR = 1/5 [TAG], as described by Friedewald et al. (30).

SEM, standard error of the mean.

^a.b,c,d^Different superscripts within a row indicate a significant difference (*p* < 0.05).

Concerning plasma metabolites, glucose levels reached the highest levels in broilers fed SPM, intermediate in SP or SPE, and lowest in broilers fed the control diet (*p* = 0.021). Urea was reduced in broilers fed SP and SPE relative to the control and SPM (*p* < 0.001). The renal function was further assessed by creatinine which displayed the highest values in broilers fed SPE and SPM (*p* < 0.001). Consistently, the SP diet increased lipid parameters which were reduced by the enzyme mixture supplementation for cholesterol (*p* < 0.001), LDL-cholesterol (*p* < 0.001), VLDL-cholesterol (*p* < 0.001), triacylglycerols (*p* < 0.001), and total lipids (*p* < 0.001). In terms of liver function, AST was reduced by Spirulina-based diets (*p* < 0.001) even if ALT was unchanged (*p* = 0.753) across dietary treatments. ALP (*p* < 0.001) and GGT (*p* = 0.002) were increased by the SP diet when compared to the other dietary treatments. Total proteins were kept unchanged across diets (*p* = 0.358). The SPE diet reduced the acute phase C-reactive protein about the other three dietary treatments (*p* = 0.002). In assessing the major electrolytes, sodium (*p* < 0.001) and chloride (*p* < 0.001) were reduced by the SPM diet relative to the other dietary treatments. Conversely, no significant changes were observed for potassium (*p* = 0.104).

### 3.3 Effect of experimental diets on hepatic total lipids, cholesterol, and fatty acid composition

The hepatic lipid profile and fatty acid composition from broilers fed 15% of *A. platensis*, either exclusively, or when Spirulina is extruded or combined with an enzyme blend (lysozyme and porcine pancreatin) are displayed in Table 4. A decline was observed in total lipids (*p* = 0.003) among broilers administered either Spirulina solely or in its extruded state, contrasting with a diminution in cholesterol levels within the control group (*p* < 0.001). Although the aggregate of saturated fatty acids (SFA) remained invariant across the dietary regimes (*p* > 0.05), certain specific fatty acids such as lauric acid (12:0) saw an elevation in levels among broilers subjected to the extruded Spirulina diet (*p* = 0.015), while palmitic acid (16.0) and arachidic acid (20.0) levels were augmented in scenarios where extruded Spirulina or Spirulina coupled with enzymes were administered (*p* = 0.009 and *p* = 0.005, respectively).

**Table 4:** Hepatic total lipids, total cholesterol and fatty acid composition of broilers fed experimental diets.

	CTR	SP	SPE	SPM	SEM	*P*-value
Total lipid (g/100 g)	2.73^a^	2.26^b^	2.34^b^	2.55^ab^	0.089	0.003
Total cholesterol (mg/g)	1.67^b^	2.47^a^	2.35^a^	2.30^a^	0.108	<0.001
Fatty acid composition (g/100 -g FA)						
12:0	0.015^b^	0.019^ab^	0.024^a^	0.018^ab^	0.002	0.015
14:0	0.24	0.20	0.22	0.27	0.024	0.337
14:1*c*9	0.015	0.018	0.021	0.025	0.004	0.259
15:0	0.047	0.058	0.054	0.054	0.004	0.269
16:0	19.5^b^	20.7^ab^	21.7^a^	21.5^a^	0.477	0.009
16:1*c*7	0.40	0.32	0.33	0.37	0.035	0.416
16:1*c*9	0.58^b^	0.98^ab^	1.09^ab^	1.19^a^	0.147	0.031
17:0	0.22	0.30	0.25	0.29	0.022	0.061
17:1*c*9	0.004	0.003	0.006	0.004	0.002	0.553
18:0	23.3	22.4	21.5	21.2	0.554	0.046
18:1*c*9	15.6^ab^	13.5^b^	15.2^ab^	18.8^a^	1.23	0.031
18:1*c*11	1.11^b^	1.44^a^	1.37^ab^	1.45^a^	0.070	0.006
18:2*n*-6	18.6^a^	17.0^ab^	16.3^b^	15.8^b^	0.563	0.006
18:3*n*-6	0.048	0.045	0.042	0.041	0.003	0.367
18:3*n*-3	0.063^c^	0.13^b^	0.20^a^	0.16^ab^	0.013	<0.001
20:0	0.086^b^	0.10^ab^	0.10^a^	0.10^a^	0.004	0.005
20:1*c*11	0.25	0.29	0.25	0.30	0.016	0.056
20:2*n*-6	0.91^a^	0.63^b^	0.54^b^	0.52^b^	0.049	<0.001
20:3*n*-6	2.19	2.56	2.40	2.33	0.150	0.371
20:4*n*-6	12.3	14.0	13.0	11.1	0.756	0.063
20:3*n*-3	0.019^b^	0.036^a^	0.040^a^	0.031^ab^	0.003	0.000
20:5*n*-3	0.039^c^	0.22^b^	0.32^a^	0.22^b^	0.017	<0.001
22:0	0.060	0.060	0.063	0.057	0.003	0.464
22:1*n*-9	0.025^b^	0.049^a^	0.035^ab^	0.031^b^	0.004	0.003
22:2*n*-6	0.015	0.023	0.030	0.019	0.006	0.257
22:5*n*-3	0.21^c^	0.56^ab^	0.69^a^	0.47^b^	0.042	<0.001
22:6*n*-3	0.52^b^	0.96^a^	0.96^a^	0.69^ab^	0.074	0.000
Others	3.58	3.34	3.16	3.04	0.247	0.442
Partial sums of fatty acids (% of total fatty acids)						
Σ SFA	43.4	43.9	44.0	43.5	0.561	0.871
Σ MUFA	18.0^ab^	16.6^b^	18.3^ab^	22.2^a^	1.41	0.047
Σ PUFA	34.9^ab^	36.2^a^	34.6^ab^	31.3^b^	1.17	0.035
Σ *n*-6 PUFA	34.1^a^	34.3^a^	32.4^ab^	29.7^b^	1.08	0.018
Σ *n*-3 PUFA	0.85^c^	1.90^ab^	2.22^a^	1.56^b^	0.105	<0.001
Nutritional ratios of fatty acids						
PUFA/SFA	0.81	0.83	0.79	0.72	0.028	0.063
*n*-6/*n*-3	41.4^a^	18.2^bc^	15.0^c^	19.4^b^	1.08	<0.001

CTR, corn and soybean meal-based diet; SP: 15% Spirulina; SPE: 15% extruded Spirulina; SPM: 15% Spirulina +0.21% Enzyme mixture (0.20% porcine pancreatin +0.01% lysozyme).

SFA = sum of (12:0, 14:0, 15:0, 16:0, 17:0, 18:0, 20:0 and 22:0).

MUFA = sum of (14:1*c*9, 16:1*c*7, 16:1*c*9, 17:1*c*9, 18:1*c*9, 18:1*c*11, 20:1*c*11 and 22:1n-9).

PUFA = sum of (18:2*n*-6, 18:3*n*-6, 18:3*n*-3, 20:2*n*-6, 20:3*n*-6, 20:4*n*-6, 20:3*n*-3, 20:5*n*-3, 22:2*n*-6, 22:5*n*-3 and 22:6*n*-3).

*n*-6 PUFA = sum of (18:2*n*-6, 18:3*n*-6, 20:*2n*-6, 20:3*n*-6, 20:4*n*-6 and 22:*2n*-6).

*n*-3 PUFA = sum of (18:3*n*-3, 20:3*n*-3, 20:5*n*-3, 22:5*n*-3 and 22:6*n*-3).

SEM - standard error of the mean.

^a.b,c^ Different superscripts within a row indicate a significant difference (*P* < 0.05).

The monounsaturated fatty acid (MUFA) aggregation peaked in broilers fed Spirulina along with the enzyme concoction, exhibited intermediate values in those fed solely Spirulina or the control diet, and plummeted to the lowest in those administered extruded Spirulina (*p* = 0.047). This pattern was predominantly driven by fluctuations in the levels of predominant fatty acid oleic acid (18:1*c*9) (*p* = 0.031), and minor fatty acids palmitoleic acid (16:1 *c*9) and vaccenic acid (18:1*c*11) (*p* = 0.006). Conversely, an inversely proportional trend was depicted in the total polyunsaturated fatty acids (PUFA) content (*p* = 0.035), and specifically the *n*-6 PUFA subcategory (*p* = 0.018), attributable largely to the proportions of linoleic acid (18:2*n*-6) (*p* = 0.006) and eicosadienoic acid (20:2*n*-6) (*p* < 0.001).

Furthermore, an elevation of total *n*-3 PUFA was noted in broilers fed extruded Spirulina, followed by those supplemented with Spirulina and enzymes (*p* < 0.001), in comparison to the control group. This ascension culminated in a notable reduction in the *n*-6/*n*-3 ratio (*p* < 0.001), particularly pronounced in the SPE diet. All individual *n*-3 fatty acids recorded an uptick under the Spirulina-based diets (*p* < 0.001) relative to the control.

### 3.4 Effect of experimental diets on hepatic diterpene profile and pigments

Table 5 shows the hepatic diterpene profile and pigment composition from broilers fed 15% of *A. platensis*, either exclusively, or when Spirulina is extruded or combined with an enzyme blend (lysozyme and porcine pancreatin). In examining the diterpene profile, β-carotene, a precursor of vitamin A, was discerned in the liver of broilers across all experimental diets, albeit with no significant changes among treatments. Conversely, a noteworthy reduction in α-tocopherol levels was observed in birds receiving Spirulina-supplemented diets (*p* < 0.001). This decrement was pronounced irrespective of whether Spirulina was extruded or complemented with the enzyme blend. Similarly, the aggregate levels of *γ*-tocopherol and β-tocotrienol exhibited a significant decrease in the Spirulina-fed treatments compared to the control (*p* < 0.001). α-Tocotrienol levels presented a marginal variance across treatments, nearing the threshold of significance (*p* = 0.052).

**Table 5:** Hepatic diterpene profile and pigments of broilers fed experimental diets.

	CTR	SP	SPE	SPM	SEM	*P*-value
Diterpene profile (μg/g)						
α-Tocopherol	23.9^a^	7.22^b^	7.09^b^	6.26^b^	0.992	<0.001
γ-Tocopherol + β-tocotrienol	0.11^a^	0.05^b^	0.06^b^	0.05^b^	0.006	<0.001
α-Tocotrienol	0.22	0.18	0.25	0.18	0.020	0.052
Pigments (μg/100 g)						
β-Carotene	n.d.	25.0	23.2	19.6	2.93	0.408
Chlorophyll-*a* (Ca)^1^	7.71^c^	34.8^a^	24.1^b^	26.5^ab^	2.28	<0.001
Chlorophyll-*b* (Cb)^2^	15.5^b^	31.6^a^	26.4^ab^	24.9^ab^	3.80	0.031
Σ Chlorophylls (Ca + b)^3^	24.4^b^	66.4^a^	50.6^a^	51.4^a^	5.43	<0.001
Σ Carotenoids (Cx + c)^4^	177^b^	1113^a^	1033^a^	958^a^	77.8	<0.001
Σ Chlorophylls and carotenoids (Ccc)^5^	201^b^	1179^a^	1084^a^	1009^a^	78.1	<0.001

CTR, corn and soybean meal-based diet; SP, 15% Spirulina; SPE, 15% extruded Spirulina; SPM, 15% Spirulina +0.21% Enzyme mixture (0.20% porcine pancreatin +0.01% lysozyme).

SEM, standard error of the mean.

n.d., not detected.

^1^Ca = 11.24 × A662 nm - 2.04 × A645 nm.

^2^Cb = 20.13 × A645 nm - 4.19 × A662 nm.

^3^Ca + b = 7.05 × A662 nm + 18.09 × A645 nm.

^4^Cx + c = (1,000 × A470 nm - 1.90 × Ca - 63.14 × Cb) / 214.

^5^Ccc = (Ca + b) + (Cx + c).

^a.b^Different superscripts within a row indicate a significant difference (*p* < 0.05).

Regarding pigments, the data manifested a marked elevation in chlorophyll-*a*, total chlorophylls, total carotenoids, and the sum of total chlorophylls plus carotenoids in the liver of broilers under all Spirulina-inclusive diets (*p* < 0.001). These enhancements were consistently, unaffected by the extrusion of Spirulina or the supplementation of the enzyme blend. Remarkably, chlorophyll-*b* levels were doubled in birds fed solely on Spirulina in comparison to those on the control diet (*p* = 0.031).

### 3.5 Effect of experimental diets on hepatic mineral profile

The mineral profile in the liver of broilers fed 15% of *A. platensis*, either exclusively, or when Spirulina is extruded or combined with an enzyme blend (pancreatin and lysozyme) is displayed in Table 6. Among the observed minerals, manganese (Mn) emerged as a distinct element displaying a significant uptick in levels in broilers nourished with either extruded Spirulina or Spirulina supplemented with the enzyme concoction, as compared to the control group (*p* = 0.008).

**Table 6:** Hepatic mineral profile of broilers fed experimental diets.

	CTR	SP	SPE	SPM	SEM	*P*-value
Macrominerals (mg/100 g)						
Calcium (Ca)	19.9	21.6	20.7	22.5	1.17	0.452
Magnesium (Mg)	23.6	22.8	22.1	23.1	0.406	0.075
Phosphorous (P)	331	319	330	321	6.81	0.523
Potassium (K)	362	350	350	352	3.89	0.120
Sodium (Na)	172	176	174	177	4.58	0.842
Sulphur (S)	213	204	207	204	3.12	0.147
Σ macrominerals	1,121	1,094	1,104	1,100	11.0	0.349
Microminerals (mg/100 g)						
Copper (Cu)	0.30	0.29	0.30	0.31	0.019	0.902
Iron (Fe)	36.6	29.4	36.6	42.4	6.98	0.631
Manganese (Mn)	0.25^b^	0.30^ab^	0.31^a^	0.31^a^	0.014	0.008
Zinc (Zn)	2.02	2.05	2.10	2.23	0.093	0.417
Σ Microminerals	39.1	32.0	39.3	45.2	6.98	0.620
Σ Macro and microminerals	1,160	1,126	1,143	1,145	13.3	0.346

CTR, corn and soybean meal-based diet; SP, 15% Spirulina; SPE, 15% extruded Spirulina; SPM, 15% Spirulina +0.21% Enzyme mixture (0.20% porcine pancreatin +0.01% lysozyme).

SEM, standard error of the mean.

^a.b^Different superscripts within a row indicate a significant difference (*P* < 0.05).

Contrary to the Mn dynamics, the remainder of the micro and macrominerals, both on an individual basis and cumulatively, maintained a steady state across all experimental factions (*p* > 0.05). The macro-mineral cadre, encompassing calcium (Ca), magnesium (Mg), phosphorous (P), potassium (K), sodium (Na) and sulphur (S), alongside the micro-mineral consortium of copper (Cu), iron (Fe) and zinc (Zn), showcased remarkable stability.

### 3.6 Principal component analysis of plasma and liver composition

**Figure 2:**
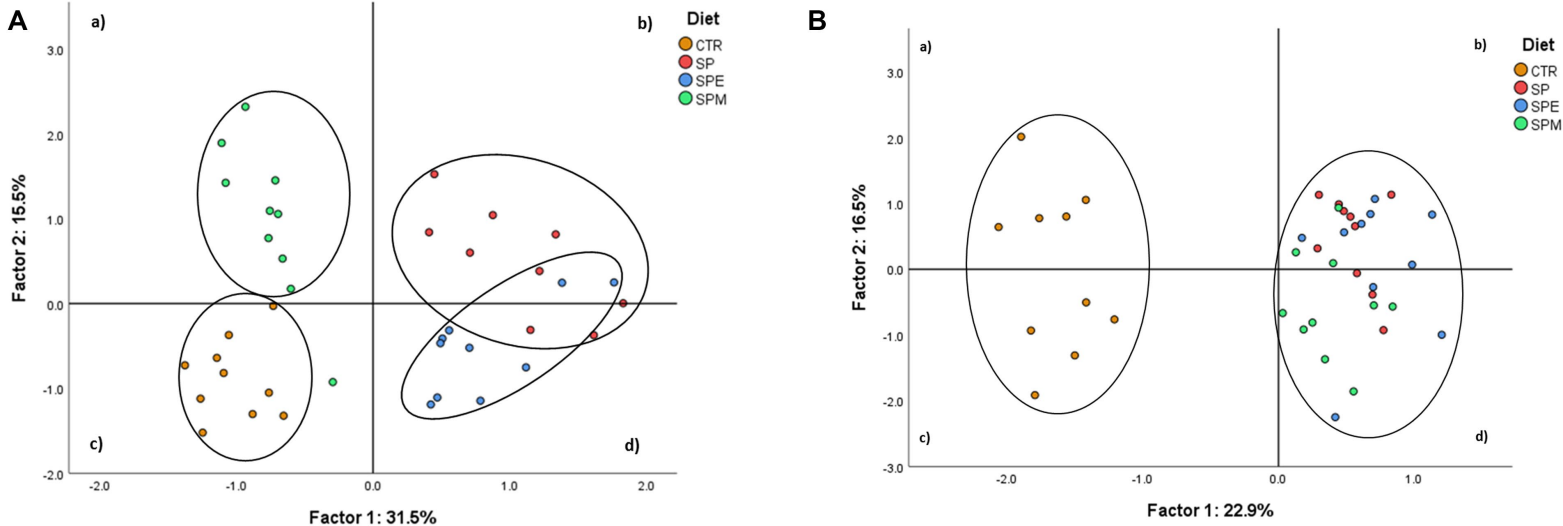
Principal component analysis of plasma metabolite profile **(A)** and hepatic biochemical composition **(B)** of dietary treatments. CTR, corn and soybean meal-based diet; SP, 15% Spirulina; SPE, 15% extruded Spirulina; SPM, 15% Spirulina +0.21% enzyme mixture (0.20% porcine pancreatin +0.01% lysozyme).

The Principal Component Analysis (PCA) was performed using variables of broilers’ plasma metabolites and hepatic lipids showed distinct variability patterns across dietary treatments (Figure 2).

Figure 2A represents the dietary treatments distributed according to the variability of plasma metabolites´ data. The first two factors covered 47.0% of the total variance (31.5% for Factor 1, and 15.5% for Factor 2) leading to a clear separation of treatments. The SPM group was mostly represented in quadrant (a), the control in quadrant (c), and SP and SPE treatments in quadrants (b) and (d). In addition, Figure 2B plots the treatments based on hepatic chemical composition. The first two factors covered 39.4% of the total variance (22.9% for Factor 1, and 16.5% for Factor 2) forming two clusters. These corresponded to the control positioned in quadrants (a) and (c), and Spirulina-containing treatments located in quadrants (b) and (d). The variables of plasma metabolites with a higher discrimination power were total lipids (0.96), LDL-cholesterol (0.92), cholesterol (0.91), VLDL-cholesterol (0.83) and triacylglycerols (0.83), for Factor 1; and HDL-cholesterol (−0.76), AST (−0.59), VLDL-cholesterol (0.45), triacylglycerols (0.45), urea (0.43), K+ (0.38) and C-reactive protein (0.33), for Factor 2 (Table 7). The hepatic variables with the most discriminant power were α-tocopherol (−0.93), 20:*5n*-3 (0.83), total carotenoids (0.81), γ-tocopherol+ β-tocotrienol (−0.78), β-carotene (0.77), chlorophyll-*a* (0.77), 22:5*n*-3 (0.75) and 18:3*n*-3 (0.74) for Factor 1; and 18:1c9 (−0.94), 20:4*n*-6 (0.87), 16:1*c*9 (−0.75), 14:1*c*9 (−0.74) and 18:0 (0.71) for Factor 2.

**Table 7:** Loadings of the first two principal components of plasma metabolites and hepatic chemical composition.

	Plasma metabolites			Liver	
Variables	Factor 1	Factor 2	Variables	Factor 1	Factor 2
Total lipids	0.96	0.18	20:5*n*-3	0.83	−0.022
LDL-cholesterol	0.92	0.06	Total carotenoids	0.81	0.08
Cholesterol	0.91	−0.11	β-Carotene	0.77	0.14
VLDL-cholesterol	0.83	0.45	Chlorophyll-*a*	0.77	−0.004
TAG	0.83	0.45	22:5*n*-3	0.75	0.45
GGT	0.60	−0.13	18:3*n*-3	0.74	0.08
Na+	0.45	−0.63	20:3*n*-3	0.66	0.38
ALP	0.39	0.11	Cholesterol	0.65	0.37
Cl−	0.35	−0.67	20:00	0.63	−0.22
Total protein	0.32	0.05	16:00	0.53	−0.54
HDL-cholesterol	0.18	−0.76	22:6*n*-3	0.53	0.63
Creatinine	0.15	0.27	18:1*c*11	0.50	−0.39
Glucose	0.021	0.12	12:00	0.48	−0.19
ALT	−0.08	0.09	16:1*c*9	0.45	−0.75
C-reactive protein	−0.29	0.33	Chlorophyll-*b*	0.45	−0.19
K+	−0.30	0.38	Manganese	0.43	0.29
AST	−0.40	−0.59	22:1*n*-9	0.42	0.27
Urea	−0.61	0.43	22:2*n*-6	0.41	−0.014
			15:00	0.34	0.44
			17:00	0.33	0.56
			14:1*c*9	0.29	−0.74
			20:1*c*11	0.24	−0.24
			20:3*n*-6	0.24	0.05
			Zinc	0.20	−0.03
			Calcium	0.18	0.10
			Sodium	0.10	0.15
			20:4*n*-6	0.068	0.87
			Copper	0.06	−0.056
			Iron	0.06	−0.30
			22:00	0.03	0.55
			17:1*c*9	0.024	0.18
			18:1*c*9	−0.001	−0.94
			14:00	−0.023	−0.57
			18:3*n*-6	−0.13	0.14
			Phosphorous	−0.19	0.01
			16:1*c*7	−0.23	−0.23
			α-Tocotrienol	−0.26	−0.29
			Sulphur	−0.32	0.29
			Potassium	−0.37	−0.22
			18:00	−0.38	0.71
			Magnesium	−0.38	0.15
			18:2*n*-6	−0.49	0.56
			Total lipids	−0.50	−0.62
			20:2*n*-6	−0.69	0.54
			γ-Tocopherol + β-tocotrienol	−0.78	0.02
			α-Tocopherol	−0.93	0.08

ALP, alkaline phosphatase (EC 3.1.3.1); ALT, alanine aminotransferase (EC 2.6.1.2); AST, aspartate aminotransferase (EC. 2.6.1.1); GGT, gamma-glutamyltransferase (EC 2.3.2.13); LDL, low density lipoproteins; TAG, triacylglycerols; VLDL, very low-density lipoproteins.

## 4 Discussion

The birds’ zootechnical data presented here resonates with the existing literature, attending that the performance of broilers is influenced by multiple factors encompassing the level of microalgae in the diet, the feeding period duration and the broilers’ efficacy in digesting microalgae’s cell walls. Park et al. (2) in a prior study illustrated that a modest dietary inclusion of *A. platensis* (ranging from 0.25 to 1.0%) over 35 days ameliorated feed efficiency in broiler chickens. Contrarily, our preceding investigation revealed a decline in final BW and BWG, along with an uptick in FCR, in birds fed 15% *A. platensis* diet, either alone or coupled with exogenous enzymes, over 21 days (3). The current endeavor, employing the identical 15% *A. platensis* dietary level, with or without commercial enzyme blends enriched with peptidases (VemoZyme P and porcine pancreatin), underscored a detrimental impact on broilers’ growth performance *post* 4 weeks of feeding, through diminished final BW, BWG and ADFI, coupled with augmented FCR. This trend was consistent throughout the experimental span, highlighting a notable impact of these diets on broiler growth performance. Nevertheless, the group fed with extruded Spirulina changed the impairment of growth performance associated with Spirulina-fed treatments leading to superior values for BW final and BWG (14 to 35d), which were equivalent to those found with the control diet. The extrusion process is not clearly understood but the disruption of microalga’s cell walls may, therefore, enhance bioavailability, with the release of important proteins, like phycocyanins. An improvement in bioavailability could facilitate more efficient hydrolysis by the broilers’ endogenous digestive enzymes, thus enhancing the absorption and consequent utilization of the nutrients, especially proteins, contributing to an improvement in broilers’ growth performance. Becker (36) propounded a cap of 10% microalgae inclusion in poultry diets to avert prospective adverse outcomes like elevated FCR over prolonged durations. The adverse bird performance with higher Spirulina levels could be ascribed to protein gelation, potentially impeding amino acid absorption and augmenting viscosity (11). Moreover, the robust protein content in Spirulina (50 to 70%) might exhibit resistance to the proteolytic action of birds’ endogenous peptidases (37). The attempt to mitigate this decline in animal growth performance via exogenous peptidase supplementation did not yield positive outcomes. A previous study in cockerels (21) also described an absence of effects on animal growth performance of a commercial super-dosed pancreatin (250 to 1,000 mg/kg) incorporated in a standard diet, although an improvement in gut morphology was detected. Conversely, studies in broilers showed a decrease in feed conversion ratio (22) and an increase in nutrient digestibility (23) when feeding similar levels (350 to 1,000 mg/kg) of a super-dosed enzyme blend added to the staple diet. However, none of the studies tested the effect of pancreatin on dietary microalgae. In the present study, the double amount of pancreatin (2000 mg/kg) was used in an attempt to maximize the hydrolysis of Spirulina compounds, but the impact of such enzyme blend on other microalgae suitable for broilers was not assessed, which would be interesting to pursue. Although *A. platensis*, being oligosaccharide-rich, is touted for its prebiotic potential which could modulate gut fermentation, this effect did not offset the adverse impact on body weight and ADG in this study; hence failing to prevent growth impairment (38). This casts light on the imperative for a more profound comprehension of dietary repercussions and nutrient utilization dynamics in broilers, to better harness the nutritional prowess of *A. platensis* while averting detrimental growth impacts.

*A. platensis* at a high level of dietary incorporation promoted no significant changes in blood cell counts, suggesting the maintenance of haematological homeostasis. This result is not aligned with Sugiharto et al. (39) findings which reported a significant reduction in haemoglobin values, erythrocytes and haematocrit in birds fed 1% of *A. platensis* during 35 days. Several studies reported an immune response balance induced by *A. platensis* (39–41). In a previous report by this research team (Lopes et al., unpublished data) using 15% of *A. platensis* in broiler chickens’ diet, we observed that while white blood cell count remained unchanged across diets, distinct variations in granulocyte and lymphocyte counts were depicted. The shift in monocyte count mirrored one of the lymphocytes. White blood cells are a part of the immune system protecting the body from infection. These cells circulate through the bloodstream and tissues to respond to injury or illness by attacking any unknown organisms that enter the body. Anomalies in their count point toward the onset of infectious or inflammatory diseases, leukaemia, lymphoma and bone marrow disorders (42). More recently, much attention has been directed to the immunomodulatory effects of Spirulina when applied as a poultry feed additive highlighting its potential to increase disease resistance and improve growth rates, particularly under stress conditions (40).

The animal’s clinical state and physical condition can be easily perceived by systemic biochemical parameters (43). In this study, we observed that the inclusion of dietary *A. platensis* at a high-level increased glucose levels in birds fed the enzyme mixture relative to the reference diet. This finding is most probably associated with the carbohydrate-rich composition of *A. platensis* (44, 45). Interestingly, while creatinine levels were enhanced by Spirulina extruded or combined with enzymes, a significant reduction in urea was observed in birds fed *A. platensis*, individual or extruded relative to the other treatments. This may suggest that *A. platensis* did not jeopardize renal function. In addition, reduced urea levels in birds fed the microalga could be a consequence of its lower protein digestibility. Often, common electrolytes such as sodium and potassium are associated with renal failure disorders (46). The kidney plays a pivotal role in maintaining the body’s chloride balance and transport, which is closely associated with sodium transport (47). Even though statistically significant, the variations observed in plasmatic sodium and chloride values, which were consistently reduced by *A. platensis* in combination with an enzyme mixture, are not relevant from a physiological point of view. In line with these findings, the element potassium did not vary among dietary treatments. Regarding the assessment of the hepatic function except for ALT, which displayed no changes across experimental treatments, the variations observed for AST, ALP and GGT enzymatic activities remained within reference figures for birds (33, 48, 49). This fact allows us to conclude that liver function is unaltered even if small changes were detected for certain aminotransferases. As so, by combining hepatic and renal data, incorporating *A. platensis* into the diets did not impose any toxicological hazard to birds underlining this microalga potential as a dietary ingredient for broiler livestock production.

The plasma lipid profile was largely influenced by diets. Our study does not support the cholesterol and lipid-lowering properties of *A. platensis* when provided individually, as previously reported for other microalgae species (50). A consistent pattern of increased total cholesterol, LDL-cholesterol, VLDL-cholesterol, triacylglycerols and total lipids was found when Spirulina was administered as an individual supplement. To the best of our knowledge, this lipemic boost is due to Spirulina addition and may be accountable to enhanced fat absorption in the intestinal tract (50). Notwithstanding, for most of these lipid parameters a counterbalanced hypolipidemic effect was detected for the combination of pancreatin with lysozyme reaching levels near to those observed in control birds. Total proteins in plasma were kept unchanged across experimental diets, even if an increase was expected in broilers fed *A. platensis* diets. Spirulina is a rich source of protein with protein levels varying from 50 to 70% (37), turning this microalga into a putative alternative for conventional protein sources, such as the traditional soybean meal (51–53). Moreover, the statistically significant differences observed for the acute phase protein, C-reactive protein, are devoid of physiological meaning because the average values found among dietary treatments were similar and followed by a residual standard error. The impacts of applying dietary *A. platensis* at a level of incorporation above 10%, as the 15% used herein, are scarce. The origin of discrepancies found between our study and the literature are determinant key factors, including dosage and source of the microalga, the experimental trial extension and specific conditions. Moreover, the main constituents of the microalgae biomass are highly dependent on microalga strain, geographical location, harvesting time of the year, and cultivation conditions (5, 28).

The liver, being the primary lipogenic tissue in poultry, is essential in shaping the fatty acid profile of broilers (54). Our experimental data delineates several nuanced interactions between the dietary inclusion of *A. platensis* and hepatic lipid profiles in broilers. The alteration in total lipids and cholesterol as observed in this study aligns with existing literature in which dietary supplementation with microalgae like Spirulina has been shown to modulate lipid metabolism in broilers, often leading to a reduction in total lipids (40). The cholesterol incremented in treatments fed Spirulina-based diets corroborates findings from other studies (3, 55). The unchanged sum of SFA across dietary treatments, despite the increment in certain individual SFAs, is an intriguing outcome. This finding is somewhat divergent from the literature in which microalgae supplementation has been associated with a decrease in SFAs due to its PUFA-rich profile (56). The elevation of MUFA in treatments fed Spirulina with an enzyme mixture aligns with findings where microalgae supplementation enhanced MUFA composition, particularly oleic acid (18:1 *c*9) (57). On the other hand, the decline in PUFA and *n*-6 PUFA in some treatments is contrary to our expectations, given Spirulina’s rich PUFA profile. The increment in *n*-3 PUFA and the reduction of the *n*-6/*n*-3 ratio, particularly in the SPE diet, is a promising finding, as a lower *n*-6/*n*-3 ratio is often deemed beneficial for health (58). This enrichment in *n*-3 PUFA, coupled with a balanced *n*-6/*n*-3 ratio, potentially augments the nutritional merit of broiler liver, which may translate to a healthier meat profile conducive to human consumption. These findings echo the complex interplay between dietary constituents and hepatic lipid metabolism, underscoring the need for a meticulous balance to optimize both animal and resultant meat health.

The increase in all individual *n*-3 fatty acids by Spirulina-based diets is a substantial finding and is supported by literature (56). The differential impacts noted between extruded Spirulina and enzyme-supplemented diets highlight the complexity of interactions between microalgae processing, enzyme supplementation, and nutrient bioavailability. Further exploration in this domain is warranted to elucidate the mechanisms underpinning these observations.

Our examination further encompassed the assessment of the impact of *A. platensis* diets, with or without enzyme supplementation, on the hepatic tocopherol and pigment profiles. A myriad of bioactive constituents originating from algae, encompassing antioxidants, pigments, vitamins, and polysaccharides, are lauded for their health-promoting attributes in both animals and humans. Spirulina, in particular, stands as an auspicious source of vitamins with potent antioxidant capabilities, known for mitigating inflammation. Vitamin E, a fat-soluble nutrient ubiquitously present in various foods, functions as a cellular antioxidant, fortifying cells against the onslaught of free radicals (59). While the dietary regime did not significantly modulate the levels of vitamin E compounds, α-tocopherol emerged as the predominant vitamin E homolog in all experimental treatments, a finding which is in harmony with the dietary composition. On the contrary, γ-tocopherol along with β-tocotrienol were discerned as minor constituents. In stark contrast, β-carotene, a precursor to vitamin A, alongside chlorophyll-*a* and total chlorophylls, witnessed a marked enhancement under the *A. platensis* diets, aligning seamlessly with its innate nutritional composition (2, 60). This unveils the bioavailability of these dietary elements and attests to the nutritional augmentation bestowed by Spirulina inclusion. Chlorophylls and carotenoids, naturally occurring lipophilic pigments, are instrumental in maintaining antioxidant homeostasis (61), thereby playing a pivotal role in both animal and human health (62). The elevations in chlorophyll and carotenoid levels in the liver as observed in this study, reflect the robust antioxidant potential promoted by Spirulina, capable of fostering a conducive antioxidant milieu in broiler chickens. This antioxidant potentiality shields the hepatic tissue from oxidative stress, which is indispensable for maintaining liver health and function. Furthermore, the unchanged tocopherol levels across the treatments underscore the stability and bioavailability of this vital antioxidant, even in the face of dietary modifications. These findings collectively accentuate the nutritional and health-promoting prowess of Spirulina, and by extension, underscore its potential as a viable, nutrient-rich feed ingredient in poultry diets.

Spirulina, like microalgae in general, embodies a rich cache of pivotal minerals (63), rendering them suitable dietary adjuncts for animals (64). Particularly, minerals like copper, iodine, iron, potassium, and zinc, which orchestrate key physiological undertakings such as cellular metabolism (e.g., iodine) and osmotic regulation (e.g., sodium), are abundant in microalgae (65). In light of this, our study ventured into discerning the impact of *A. platensis* diets on the hepatic mineral terrain of broilers. As showcased in this study, the overall concourse of macrominerals sustained a level of constancy across the Spirulina dietary spectrum, albeit sodium and magnesium manifested divergent trajectories. The hepatic sodium reservoir experienced an uptick in birds nurtured on Spirulina diets, marking a positive stride. Sodium, an indispensable nutrient, plays an important role in cellular homeostasis, fluid and electrolyte equilibrium, and blood pressure modulation. Additionally, it is instrumental in orchestrating muscle and nerve cell excitability alongside facilitating the transit of nutrients and substrates across plasma membranes (66). Conversely, despite a diminution in magnesium levels in birds fed on *A. platensis* diets, the decrement was marginal. Magnesium, a versatile mineral, serves as a cofactor for an expanse of over 300 enzymes, thereby modulating crucial physiological activities like muscle contraction, neuromuscular conduction, glycaemic control, myocardial contraction, and blood pressure regulation (67). On the flip side, the infusion of *A. platensis* with enzyme supplementation did not elicit any discernible alterations in micromineral levels.

Microminerals, such as zinc, manganese, and copper, are esteemed as quintessential cofactors for antioxidant enzymes like superoxide dismutase (68) and constitute the vanguard of antioxidant defence. Iron, a life-essential metallic entity, is implicated in engendering deleterious oxygen species via the Fenton reaction, culminating in the genesis of the potent hydroxyl radical. The transport, utilization, and storage of iron are meticulously carried out by specialized proteins like transferrin, ferritin, and haemoproteins (69, 70). The increase in hepatic Mn accentuates the potential role of dietary Spirulina in enhancing the bioavailability or retention of certain trace minerals, thus possibly fortifying the mineral nutritional status of the broilers. Collectively, the micromineral findings echo the maintenance of the redox balance within the birds, potentially underpinning a state of antioxidant homeostasis. This narrative dovetails with the overarching narrative of microalgae, especially Spirulina, being a nourishing source of essential minerals, thereby potentially fortifying the mineral nutritional status of broilers and by extension, showcasing its promise as a robust dietary supplement in poultry nutrition.

The results of the PCA align with prior studies (33, 71, 72), effectively demonstrating the distinct impacts of the experimental diets on plasma metabolites and hepatic variables in broilers. The PCA revealed four distinct clusters corresponding to each treatment group, with notable overlap between the SP and SPE treatments, yet a clear demarcation of the SPM and control treatments. This pattern suggests a significant influence of the enzyme mixture on key discriminant variables such as total lipids, triacylglycerols, and cholesterol. However, these PCA findings do not completely mirror the data presented in Table 3, indicating a complex interaction between diet composition and metabolic outcomes. The differential impact of enzyme supplementation in diets containing microalgae, observed in this study, echoes previous findings where the inclusion of 0.001% of carbohydrases, either a four-enzyme moisture (exo-β-glucosaminidase, alginate lyase, peptidoglycan N-acetylmuramic acid deacetylase and lysozyme) (NzyTech, Lisbon, Portugal) or a PL25 ulvan lyase (NzyTech, Lisbon, Portugal) feed combined with 10% of *Chlorella vulgaris* (33) and 15% of *Ulva lactuca* (71), respectively, altered the nutritional dynamics in broiler diets. Additionally, the PCA’s liver variable analysis distinguished control treatments from those fed microalgae, a finding consistent with Costa et al. (72), who reported similar differentiation in diets supplemented with 15% of *Laminaria digitata* and 0.001% of alginate lyase. The distinct power of Spirulina is attributed to the changes in key discriminant variables like certain *n*-3 PUFAs (18:3 *n*-3, 20:5 *n*-3, and 22:5 *n*-3), α- and γ-tocopherols, β-carotene, chlorophyll-*a*, and total carotenoids. These observations corroborate the findings presented in Tables 4, 5, which showed an increase in these fatty acids and pigments and a decrease in vitamin E levels with the inclusion of microalgae. Although Altmann et al. (73) also showed a distinct impact of Spirulina for *n*-3 PUFA, the results obtained were opposite relative to those presented in this study, which might be attributed to the different nutritional composition of microalga. Collectively, these results underscore the capacity of Spirulina, particularly when combined with enzymatic supplementation, to modulate plasma lipid components. Additionally, the data demonstrate the effect of the microalga alone in enhancing liver lipid metabolism and composition, primarily through the accumulation of health-beneficial *n*-3 PUFAs and antioxidant pigments. Further dissemination of the present results through social media, evidencing the benefits and constraints of incorporating Spirulina in broiler’s diet to the general public represents a promising prospective for spreading scientific knowledge (74).

## 5 Conclusion

This study elucidates the multifaceted impact of 15% dietary inclusion of Spirulina in broiler chickens, highlighting alterations in systemic antioxidant capacity, hepatic fatty acid composition and growth performance. Specifically, the extruded and enzyme-supplemented Spirulina diets significantly elevated hepatic *n*-3 fatty acid levels, fostering a favorable shift in the *n*-6/*n*-3 PUFA ratio, which is indicative of potential health benefits. This inclusion level of Spirulina also led to a reduction in total hepatic lipids and increased antioxidant carotenoids in the broiler’s liver. However, the hepatic cholesterol was increased and α-tocopherol was decreased by the addition of Spirulina, which was not reverted by either the extrusion pre-treatment or the enzymatic supplementation. The incorporation of microalga was also responsible for an increase in total cholesterol, LDL-cholesterol and total lipids in the blood, although the enzymatic mixture counteracted this effect. The microalga had adverse effects on growth performance, which were not mitigated by the enzyme supplementation but partially reverted by microalga extrusion. The extrusion of Spirulina appeared to enhance the bioavailability of some nutrients, signifying a potential processing avenue to maximize the nutritional benefits of Spirulina. These findings underscore a complex interplay between dietary Spirulina inclusion, its processing, enzymatic supplementation and the resultant effects on broiler health and growth performance.

Future studies should attempt to delineate the optimal inclusion levels and processing methods for Spirulina to mitigate the adverse effects on cholesterol and α-tocopherol, and, thus, maximizing health benefits. Additionally, the precise mechanisms underlying the observed effects, particularly the interaction between Spirulina processing, enzyme supplementation and nutrient bioavailability warrant in-depth investigation. The extrusion pre-treatment of microalga shows potential for application up to an industrial scale. Therefore, extending the research to include evaluations of the long-term effects of dietary Spirulina inclusion, and exploring varying levels of enzymatic supplementation combined with pre-extruded alga may provide more nuanced insights into harnessing the potential benefits of Spirulina in poultry diets.

## Data availability statement

The raw data supporting the conclusions of this article will be made available by the authors, without undue reservation.

## Ethics statement

The investigation strictly complied with the regulatory framework stipulated by the European Union (Directive 2010/63/EU) and procured requisite endorsements from the Ethics Review Board of CIISA/FMV, the Animal Welfare Committee of the National Veterinary Authority (Direção Geral de Alimentação e Veterinária, Portugal), and ORBEA/ISA (approval code: 0421/000/000/2022).

## Author contributions

MS: Writing – review & editing, Investigation, Formal analysis, Methodology. CA: Writing – review & editing, Writing – original draft. MC: Writing – review & editing, Writing – original draft, Validation, Investigation, Formal analysis, Methodology. RP: Writing – review & editing, Investigation, Methodology. PL: Writing – review & editing, Writing – original draft. JPe: Writing – review & editing, Investigation, Methodology. JT: Writing – review & editing, Investigation. AM: Writing – review & editing, Investigation. MM: Writing – review & editing, Investigation, Methodology. BT: Writing – review & editing, Investigation. DC: Writing – review & editing, Investigation, Methodology. CM: Writing – review & editing, Investigation, Methodology. JF: Writing – review & editing, Investigation. ML: Writing – review & editing, Resources. JPr: Writing – review & editing, Writing – original draft, Validation, Supervision, Resources, Project administration, Funding acquisition, Conceptualization.
